# Belatacept associated - cytomegalovirus retinitis in a kidney transplant recipient: a case report and review of the literature

**DOI:** 10.1186/s12886-020-01741-1

**Published:** 2020-12-01

**Authors:** Pierre-Guillaume Deliège, Justine Bastien, Laetitia Mokri, Charlotte Guyot-Colosio, Carl Arndt, Philippe Rieu

**Affiliations:** 1 Division of Nephrology, University Hospital of Reims, Reims, France; 2 Division of Ophtalmology, University Hospital of Reims, Reims, France; 3 Laboratory of Nephrology, UMR CNRS URCA 7369 (Matrice Extracellulaire et Dynamique Cellulaire, MEDyC), Reims, France

**Keywords:** Cytomegalovirus, CMV, Retinitis, Kidney transplantation, Belatacept

## Abstract

**Background:**

To report the first case of belatacept-associated multidrug-resistant Cytomegalovirus retinitis in a kidney transplant recipient.

**Case presentation:**

A 76-year-old African male renal allograft recipient was admitted for acute visual loss of the right eye. Ophthalmological examination of the right eye showed anterior uveitis and vitritis associated with large paravascular haemorrhages and yellow necrotic borders, involving the posterior pole but not the fovea. Both Cytomegalovirus DNA in plasma and aqueous humor were positive. The patient had had several episodes of Cytomegalovirus reactivation subsequent to the introduction of belatacept. His cytomegalovirus was multi-drug resistant, and was treated with maribarir, intravitreal and systemic injections of foscarnet, and anti-Cytomegalovirus human immunoglobulin. In parallel, belatacept was stopped and switched to tacrolimus. Cytomegalovirus DNA became undetectable and there was partial improvement of visual acuity at the last ophthalmologic examination, 18 months after the initial diagnosis of Cytomegalovirus retinitis.

**Conclusion:**

Cytomegalovirus retinitis is an uncommon opportunistic infection in kidney transplant recipients. Cytomegalovirus retinitis is a serious infection because of the risk of blindness and the occurrence of associated life-threatening opportunistic infections. In view of the recent literature, kidney transplant recipients treated by belatacept immunosuppression may be at increased risk for Cytomegalovirus disease, notably Cytomegalovirus retinitis. The occurrence of Cytomegalovirus retinitis may help improve the selection of patients converted to belatacept.

## Background

Cytomegalovirus (CMV) is the most frequent pathogen after kidney transplantation. It occurs mainly during the first 6 months after transplantation, secondary to primo-infection (transmission from the donor organ), reactivation of latent infection or reinfection with a different strain [1]. CMV replicates intracellularly; the immune response is therefore cellular-mediated, by CMV-specific CD4 and CD8 T-lymphocytes [1, 2].

CMV infection (detection of CMV virus or viral proteins in blood or any secretion) can result in CMV disease with a range of clinical manifestations from mild viral syndrome to encephalitis, gastro-intestinal disease, hepatitis, myocarditis, pancreatitis, pneumonia, nephritis, retinitis [1].

CMV retinitis is a rarely encountered complication in kidney transplant recipients, in contrast to its frequency in HIV-infected patients. Akova et al. described only one case of CMV retinitis in a monocentric retrospective study over a ten-year period, involving 1156 patients who underwent renal transplantation [3]. Eid et al. conducted a retrospective review of all cases of CMV retinitis after solid organ transplantation at the Mayo Clinic during 1990–2004: during this period, only five cases of CMV retinitis were observed after kidney transplantation [4]. In parallel, a few case reports/series of CMV retinitis have been published in kidney transplant recipients [5–13].

Given the development of belatacept, an immunosuppressive therapy acting as a selective T-cell co-stimulation blocker, and the fact that the immune response against the virus is cellular mediated by CMV-specific T-lymphocytes, it is necessary to identify new potential side effects of belatacept.

## Case presentation

A 76-year-old African male renal allograft recipient with diabetes mellitus and hypertension, was presented to the hospital with a 14-day history of reduced vision in the right eye.

He had received a first kidney transplant in 1995 for end-stage renal disease due to unknown nephropathy. He also had a history of malarial splenomegaly, with a splenectomy in 1992. During his first transplant, the patient developed CMV primo-infection.

He then returned for hemodialysis on 12/2003 following the deterioration of renal function in connection with episodes of acute cellular rejection and an allograft glomerulopathy confirmed by histological analysis. Transplantectomy was performed in October 2004 because of abdominal pain and hematuria.

In September 2009, he received a deceased donor renal transplant. The donor was a 64-year-old woman, with a history of auto-immune cirrhosis treated by tacrolimus. Induction therapy consisted of basiliximab and maintenance immunosuppression, associated with tacrolimus, mycophenolate mofetil, and methylprednisolone.

The CMV and the EBV serostatus at the second transplant were respectively donor (+) /recipient (+) and donor (+)/recipient (+) and he received prophylactic low-dose valganciclovir for 4 months after transplantation. CMV DNA detection, assessed twice on 02/2010, was negative (< 1000 copies/ml).

Three months after transplantation, the graft function was impaired (serum creatinine level 320 μmol/L), and graft biopsy revealed tacrolimus toxicity and nephroangiosclerosis, without rejection. Tacrolimus was switched to sirolimus on 02/2010.

On 02/2012, sirolimus was switched to belatacept (5 mg/kg day 0, day 15, day 30, then every month) because of the occurrence of sirolimus-induced bronchiolitis obliterans organizing pneumonia.

After 2012, several CMV reactivations occurred, treated by sequential valganciclovir 450 mg/day corrected by GFR (stopping valganciclovir after two negative CMV DNA). Methylprednisolone and mycophenolate mofetil were respectively lowered to 5 mg and 600 mg × 2/day. CD4 T-lymphocyte count was below 500 cells/μL.

On 11/2017, CMV reactivation occurred despite permanent prophylaxy (valganciclovir 450 mg twice a week corrected by GFR) having been introduced in 2016.

**Fig. 1:**
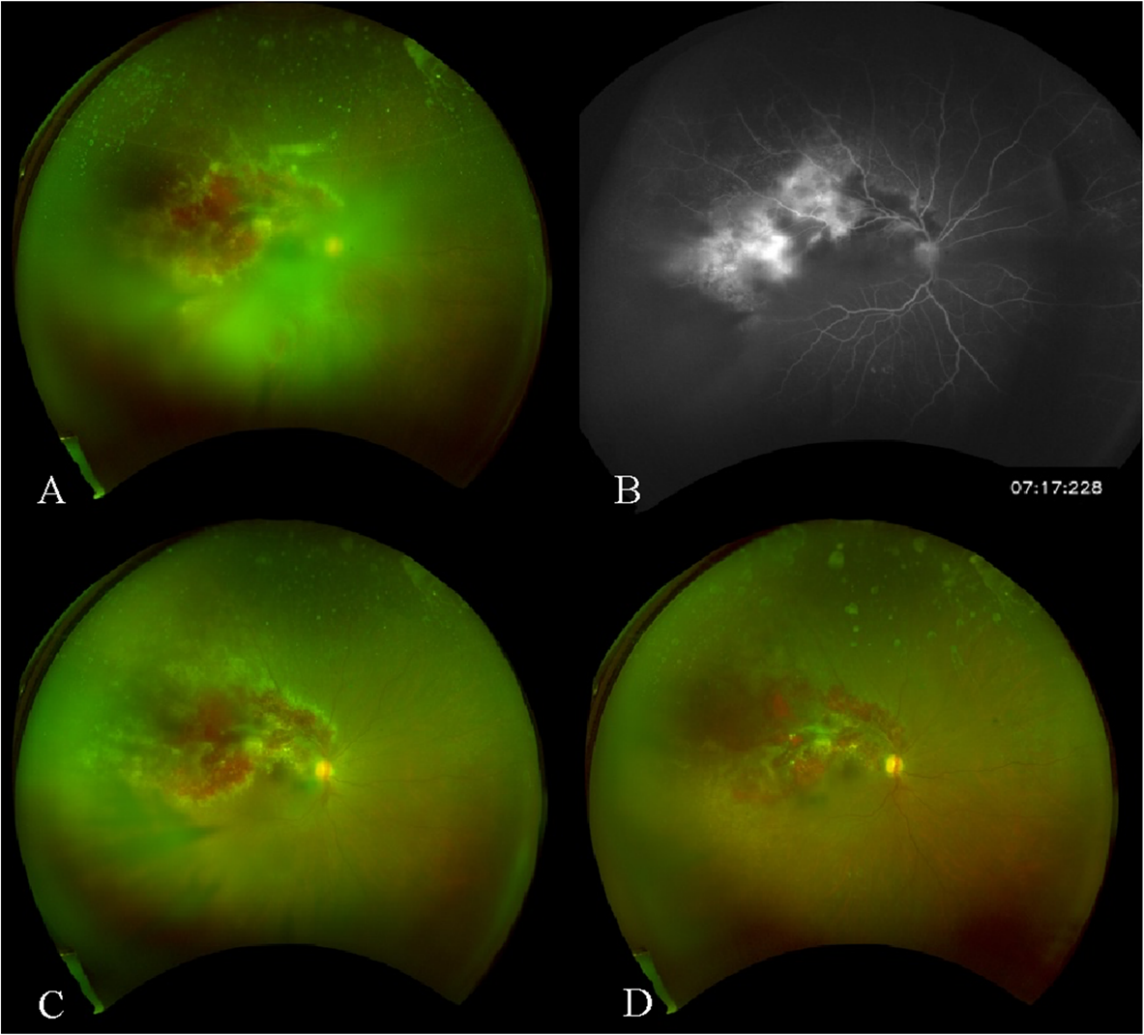
**a**: Color fundus photograph of the right eye showing inflammation of the posterior chamber (vitritis) associated with large paravascular haemorrhages and yellow necrotic borders. **b**: Fluorescein angiography of the same eye showing hypofluorescence due to hemorrhages, and hyperfluorescence due to leakage. **c**: Evolution after 2 weeks: attenuation of vitritis, stabilization of retinitis. **d**: Evolution after 1 month: resolution of vitritis, stabilization of retinitis

He was then admitted with the chief complaint of decreased visual acuity in the right eye. Best corrected visual acuity (VA) was 20/200 in the right eye and 20/20 in the left eye. Intraocular pressure was 10 mmHg in the right eye and 18 mmHg in the left eye. Slit-lamp and fundoscopic examination of the right eye showed respectively anterior uveitis rated to 2+ for both anterior chamber cells and flare, according to the Standardization of Uveitis Nomenclature (SUN) guidelines and vitritis associated with large paravascular haemorrhages and yellow necrotic borders. The retinal lesion was located on the temporal superior vascular arcade and involved the posterior pole but not the fovea, which was confirmed by angiography (Fig. 1a and b). The left eye appeared normal. Both CMV DNA in serum and aqueous humor were positive (respectively 330,000 copies/mL and 76,000 copies/mL). There was no other tissue-invasive disease and no acute graft dysfunction.

Systemic Ganciclovir (5 mg/kg/12 h) was ineffective and CMV DNA increased to 550,000 copies/mL. Analysis confirmed the presence of ganciclovir and cidofovir-resistant CMV, due to UL97 mutation (His520Gln) and UL54 mutation (Pro522Ser). He received an intravitreal injection (IVT) of foscarnet (6 g/250 mL; 0.1 mL twice a week over 2 weeks, then 1 per week over 6 weeks), associated with systemic maribavir 500 mg twice daily and anti-CMV human immunoglobulin (100 U/mL; 4 mL/kg at J0, J4 and J8, then 2 mL/kg at J12 and J16, then maintenance therapy 1 mL/kg every 2 weeks). Surgery was not retained. It was decided to stop belatacept and mycophenolate mofetil, and switch to tacrolimus, in association with methylprednisolone 15 mg/day.

Slit-lamp examination showed the disappearance of the anterior uveitis within 1 week. Fundoscopic examination showed progressive resorption of the vitritis, and no change in the retinitis (Fig. 1c and d). After cessation of IVT foscarnet, CMV retinitis appeared again with a new supra-papillary focus, although CD4 T-lymphocyte count was above 500 cells/μL and CMV QuantiFERON showed lymphocyte reactivity against CMV. New resistance to maribavir was detected: UL54 mutation (D515Y). It was then decided to switch maribavir to systemic foscarnet (15 mg/kg/day for 1 month in July 2018), despite chronic graft malfunction. CMV DNA in the serum stabilized between 1000 and 6000 copies/mL and then became undetectable in 12/2018. Anti-CMV human immunoglobulin (1 mL/kg/2 weeks) was stopped in 04/2019. At the last ophthalmologic examination in 09/2019, best corrected VA was 20/30 in the right eye and 20/40 in the left eye. Intraocular pressure was 15 mmHg in the right eye and 13 mmHg in the left eye. Slit-lamp and fundoscopic examination of the right eye showed, respectively, quiet anterior chamber and sequellar pigmented retrodescemetic precipitates. The left eye presented with a corticonuclear cataract. CMV DNA in serum was undetectable, in the absence of CMV specific treatment and with immunosuppressive therapy of tacrolimus and methylprednisolone 10 mg/day.

## Discussion and conclusion

To our knowledge, this is the first multidrug-resistant CMV retinitis case associated with belatacept as maintenance immunosuppressive therapy in a kidney transplant recipient.

CMV infection is the most frequent opportunistic infection after kidney transplantation. Risk factors affecting the incidence of CMV disease are donor and recipient serostatus (CMV-seronegative recipients of CMV-seropositive donors [D+/R-] are at the highest risk), type and dosage of immunosuppressive drugs (induction, cyclosporine use), donor age (over 60 years old), simultaneous kidney-pancreas transplantation, presence of acute rejection episodes, and chronic graft malfunction [1, 14]. Risk factors associated with the occurrence of CMV retinitis are having a CD4 T-lymphocyte count below 50 cells/μL at the prior visit in HIV-infected patients and a peak CMV DNA level superior to 76,400 copies/mL after hematopoietic stem cell transplantation. Long duration of CMV viremia is associated with CMV retinitis only in univariate analysis [15, 16]. For CMV retinitis after kidney transplantation, few cases have been reported in the literature and available data is limited. It is therefore difficult to identify risk factors [5–12]: it affects middle-aged men and women, usually under maintenance immunosuppressive tritherapy including calcineurin inhibitors (CNI). Median time from the date of transplantation to diagnosis of CMV retinitis was 9 months (range, 3 months to 13 years). CMV retinitis manifests with decreased visual acuity, floaters, scotomas. However CMV retinitis can also be asymptomatic and under-recognized. Funduscopic examination often shows bilateral necrotic retinitis, associated with uveitis. CMV viremia is not always contributive. CMV detection in aqueous humor differentiates active versus inactive retinitis. Systemic and IVT ganciclovir (or valganciclovir if available) is the first-line treatment for retinitis, and prolonged therapy can result in the development of resistance due to mutations in the viral phosphotransferase (UL97 gene) and/or in the viral DNA polymerase (UL54 gene). For CMV retinitis in kidney transplant recipients, there have been three cases indicating multidrug-CMV resistance [5, 7, 13]. A complete resolution can be observed, if the diagnosis and treatment are given early, or there can be recurrence, sequellar visual loss or death due to associated life-threatening opportunistic infections, such as nocardiosis or aspergillosis. Our patient had a history of diabetes mellitus, splenectomy, and a previous transplant. The donor was 64 years old. He also had a low count of CD4 T-lymphocytes but more than 50 cells/μL, a high peak of CMV DNA (550,000 copies/mL on 11/2017) and long-term CMV viremia (at least 30 months). CMV retinitis occurred 102 months after transplantation. He was under immunosuppressive tritherapy including belatacept in place of CNI.

There is insufficient data on the safety of beletacept in relation to CMV infection. Bassil et al. did not find a difference in the rate of CMV viremia between belatacept- and CNI-based regimen in his monocentric 36-month study [17]. The seven-year BENEFIT (Belatacept Evaluation of Nephroprotection and Efficacy as First-line Immunosuppression Trial) study suggests that CMV diseases tend to be more frequent in belatacept groups (respectively 1.4 and 1.1 number of events/person-year in more intensive and less intensive belatacept groups versus 0.8 number of events/person-year in cyclosporine group) [18]. Grinyo et al. showed more CMV viremia 3 years after switching CNI to belatacept, but never serious diseases according to MedDRA [19]. No new safety signals emerged for belatacept with up to 10 years of exposure [20] and there is no specific data on CMV retinitis in these different studies. Very recently, Fan et al. described two cases of CMV retinitis with belatacept immunosuppression [21] and Bertrand et al. reported a significant risk of CMV disease post-conversion to belatacept, notably two cases of CMV chorioretinitis, an « exceptional finding in kidney transplantation », in a multicentric French cohort of 280 kidney transplant patients [22].

In our case, after the introduction of belatacept, the patient presented several CMV reactivations, then developed a CMV multidrug-resistant infection with active retinitis. The occurrence of CMV retinitis may contribute to the better selection of patients converted to belatacept. Further studies are needed to better identify risk factors for CMV retinitis in kidney transplant recipients.

## Data Availability

The data that support the findings of this study are available upon request from the authors.

## Abbreviations

CD: Cluster of differentiation
CMV: Cytomegalovirus
CNI: Calcineurin inhibitors
EBV: Epstein Barr Virus
HIV: Human Immunodeficiency Virus
IVT: Intravitreal injection
MedDRA: Medical Dictionary for Regulatory Activities
VA: Visual acuity
